# Mechanochemically assisted hydrolysis in the ADOR process†
†Electronic supplementary information (ESI) available. See DOI: 10.1039/d0sc02547j


**DOI:** 10.1039/d0sc02547j

**Published:** 2020-06-15

**Authors:** Daniel N. Rainer, Cameron M. Rice, Stewart J. Warrender, Sharon E. Ashbrook, Russell E. Morris

**Affiliations:** a School of Chemistry , EaStCHEM , University of St. Andrews , North Haugh, St. Andrews , Fife , KY16 9ST , UK . Email: rem1@st-andrews.ac.uk; b Department of Physical and Macromolecular Chemistry , Faculty of Sciences , Charles University , Hlavova 8 , 128 43 Prague 2 , Czech Republic

## Abstract

The ADOR (Assembly-Disassembly-Organisation-Reassembly) process for zeolites has been shown to produce a number of previously unknown frameworks inaccessible through conventional synthesis methods. Here, we present successful mechanochemically assisted hydrolysis of germanosilicate zeolite **UTL** leading to ADOR products under mild conditions, low amounts of solvent and in short reaction times. The expansion of zeolite synthesis into the realm of mechanochemistry opens up feasible pathways regarding the production of these materials, especially for industrial purposes, as well as an exciting application for economical enrichment of materials with the low natural abundance NMR-active isotope of oxygen, ^17^O. The results from mechanochemically assisted hydrolysis differ from those seen in the traditional ADOR approach: differences that can be attributed to a change in solvent availability.

## Abstract 2

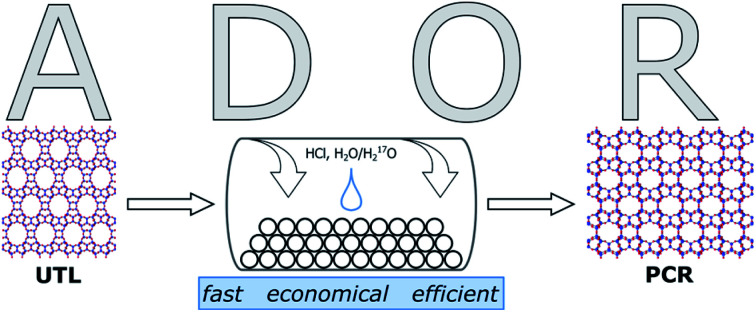
Efficient hydrolysis of zeolites in the ADOR process using mechanochemistry, including economical enrichment with ^17^O for solid-state NMR.

## Introduction

### Germanosilicate zeolites and the ADOR process

**Fig. 1:**
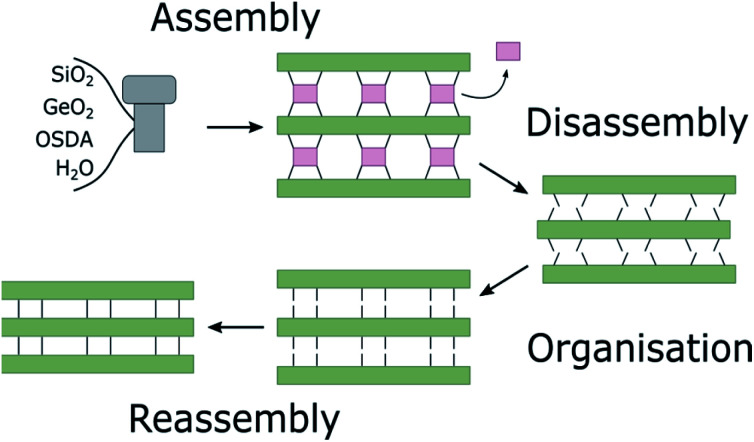
Schematic representation of the ADOR process. Assembly of parent zeolite *via* hydrothermal synthesis. Disassembly by hydrolysis to form the layered precursor. Organisation of layered intermediate by intercalation with inorganic or organic compounds. Reassembly by thermal treatment to form fully connected daughter zeolite.

Zeolites are microporous inorganic materials, built up from tetrahedrally connected [TO_4_] units (T = Si, Al, Ge, B, …).^1^–^3^ They have been used for a wide variety of industrial applications, ranging from catalysis^4^,^5^ to ion exchange,^6^ gas storage,^7^ and many more. Over the last years, germanosilicate zeolites have been established as promising materials, due to their extra-large pore containing frameworks.^8^–^11^ An even more recent development exploits the instability of the germanium–oxygen bond regarding hydrolysis in the so-called ADOR process.^12^–^14^ In the ADOR process the conventionally assembled germanosilicate (generally *via* hydrothermal synthesis) is successively disassembled (hydrolysis by water/acid), organised, if necessary by means of a structure-directing agent (organic or inorganic), and finally reassembled by thermal treatment. A schematic illustration of the ADOR process is given in Fig. 1. The success of this procedure is based on the defined location of the germanium atoms in the parent zeolite. They preferentially occupy positions in cubic double four-ring (d4r) units,^15^,^16^ enabling selective targeting of this compositional building block.

**Fig. 2:**
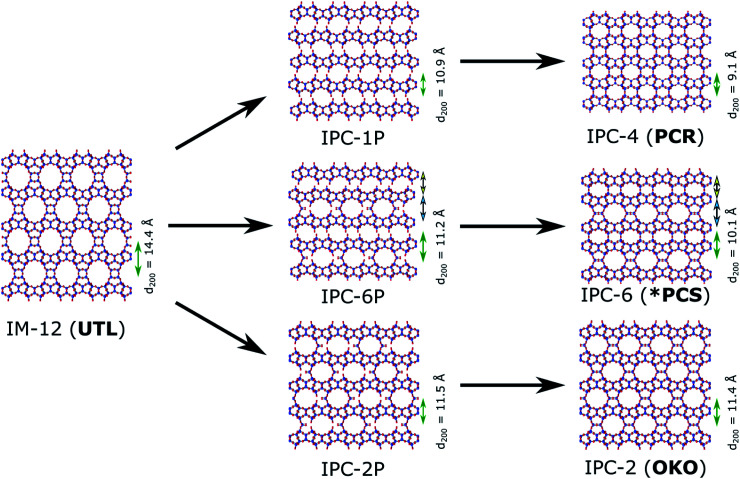
Zeolite **UTL**, its hydrolysis products IPC-1P, IPC-6P and IPC-2P and final new zeolites IPC-4 (**PCR**), IPC-6 (*PCS) and IPC-2 (**OKO**). All frameworks are shown in (001) direction, and the *d*-spacings for the (200) plane are given in Å and marked by green arrows. The values for *d*_200_ of IPC-6P and IPC-6 are calculated as the average of the two interlayer distances, indicated by blue and yellow arrows.

In a zeolite such as IM-12 (with framework topology code **UTL**), where d4rs can be viewed as linkages between layers, selective removal of the connecting cubic units leaves the almost purely siliceous layers intact. In the case of **UTL**, the layered product of this selective hydrolysis has been named IPC-1P.^12^ The individual layers can be arranged in various ways to form new zeolites, as shown in Fig. 2. Direct connection of the layers *via* bridging oxygen atoms leads to IPC-4 (assigned the zeolite framework code **PCR**).^17^ It is also possible to reintroduce silicon between the layers as square s4r units, either under highly acidic hydrolysis conditions (*e.g.* 12 M hydrochloric acid, HCl) or through the addition of a secondary silicon precursor such as diethoxydimethylsilane, resulting in IPC-2 (**OKO**).^13^,^18^,^19^ Using 6 M hydrochloric acid directs the formation of IPC-6 (*PCS),^20^ an intermediate between IPC-2 and IPC-4, comprised of half **OKO**-type and half **PCR**-type linkages. Furthermore, so-called “unfeasible” zeolites IPC-9 and IPC-10, containing odd-numbered rings in their framework, were produced using choline cations as intercalation agents, which changes the relative alignment of the layers before reassembly.^21^

The transformation of the parent material **UTL** into its corresponding ADOR daughter zeolites can be followed by powder X-ray diffraction (PXRD).^22^,^23^ The position of the peak originating from the crystallographic (200) plane, usually the dominant feature in ADOR-derived zeolites, corresponds to the spacing between the siliceous layers of the framework. A shift towards higher angles, while retaining intralayer reflection positions, indicates removal of the connecting units and production of a disassembled intermediate. Depending on the actual 2*θ* value, conclusions about the nature of the linkages are possible.^22^

Zeolites with frameworks **IWR**,^24^**IWW**,^25^,^26^ **UOV**,^27^,^28^ and *CTH^29^ exhibit similar structural features as **UTL** in that they contain d4r units, and have been successfully used in the ADOR process to produce a variety of new frameworks. There are still more frameworks to explore, such as zeolite **IWV**,^30^,^31^ which also contain the necessary structural features required for ADOR.

### Mechanochemical methods in zeolite chemistry

One of the goals of modern chemistry is improvement in terms of atom efficiency and effectiveness in syntheses. This can be achieved by designing the synthesis process itself to be more energy efficient or reducing the solvent volumes required for a given reaction. A promising methodology is mechanochemistry, applicable in different fields of chemistry, as shown in various literature reviews.^32^–^41^

The applicability and importance of mechanochemical methods for zeolite chemistry has recently been outlined by Pérez-Ramírez and co-workers^42^ as well as Morris and James.^43^ Reducing the amount of solvent (solvent-assisted methods) or even omitting it altogether (solvent-free approaches) could lead, and in some cases already has led, to improvements in terms of the quantity of waste products and in energy efficiency, resulting in an overall more economical process.^44^,^45^ Additionally, mechanochemical treatment has been shown to yield zeolite catalysts with good performance, for example through simultaneous particle size reduction during alumination.^46^

The established knowledge about the ADOR process has been compiled in a recent review,^47^ yet further insight into the underlying fundamental principles is necessary. In particular, the disassembly step is currently being investigated,^22^,^23^,^48^ as it is probably the most crucial of the entire process. Previous work has also been concerned with the effect of high pressure on the final, reassembly step.^49^ Additionally, recovery of germanium has been reported to be a viable strategy for improving the feasibility of germanosilicate zeolite synthesis.^50^

The aim of the present work is to study how additional mechanical forces may influence the hydrolysis (disassembly) step of the ADOR process, and to explore possibilities for more economical synthesis routes, particularly of interest for potential scalability.

The study highlights the feasibility of an alternative synthesis method based on mechanochemical ball milling for the generation of known ADOR zeolites. In addition, unexpected products were obtained in this process compared to the conventional synthesis. Notably, when using hydrochloric acid IPC-2 is normally the favoured product, but in mechanochemical synthesis IPC-4 with a denser framework was obtained. This different mechanism may open up new possibilities for the ADOR process, particularly when considering the ease of scalability of ball milling processes, and in the isotopic enrichment of the material with expensive ^17^O using small amounts of liquid.

Studying the oxygen environment of solids with nuclear magnetic resonance (NMR) spectroscopy can yield valuable insight into the structure of inorganic materials. However, ^17^O NMR has not yet found widespread use due to the low natural abundance levels of this isotope (0.037%), and the additional line broadening that results from its quadrupolar nature (*I* = 5/2). Enrichment protocols, aiming to increase the amount of ^17^O in the sample, typically suffer from the cost of the enriched reagents required and cumbersome adaptions to the conventional syntheses to ensure atom-efficient incorporation of the isotopic label. For microporous materials, great progress has been made in this respect using a low volume hydrolysis approach in the ADOR process,^51^ ionothermal synthesis,^52^ dry gel conversion reactions^53^,^54^ and H_2_^17^O steaming of MOFs.^54^ The applicability of mechanochemical ball milling for enrichment has been demonstrated by Laurencin and co-workers on several metal oxides.^55^,^56^ The results presented here demonstrate another example of the successful employment of mechanochemistry for this isotopic enrichment with levels of isotope incorporation comparable to those published previously for ADOR derived materials.^51^

## Experimental

### Chemicals

Cab-O-Sil M5 (Acros Organics), germanium dioxide (Sigma-Aldrich, 99.998%), *cis*-2,6-dimethyl piperidine (Sigma-Aldrich, 98%), 1,4-dibromobutane (Sigma-Aldrich, 99%), potassium carbonate (Fisher Scientific), acetonitrile (Fisher Scientific), diethyl ether (Honeywell), ethanol (VWR), Ambersep 900(OH) ion exchange resin (Alfa Aesar), hydrochloric acid (Fisher Scientific), 40% H_2_^17^O (CortecNet).

### Synthesis of parent zeolite IM-12 (**UTL**)

#### Synthesis of the structure-directing agent

The synthesis of the organic structure directing agent (OSDA) was performed by adapting a procedure by Marino *et al.*^57^ 0.5 mol 1,4-dibromobutane (1 eq., 108 g), and 0.6 mol potassium carbonate (1.2 eq., 83 g) were placed in a round bottom flask together with 500 ml acetonitrile as solvent. 0.5 mol *cis*-2,6-dimethyl-piperidine (1 eq., 56.6 g) were added dropwise under stirring, followed by heating (under continuous stirring) at 90 °C overnight. Acetonitrile was removed under reduced pressure, the solid dissolved in ethanol and the carbonate removed by filtration. Ethanol was removed to produce a saturated solution and the final bromide salt is reprecipitated by addition of diethyl ether. The white crystals were recovered by filtration and dried *in vacuo*. The purity was confirmed by ^1^H- and ^13^C-NMR (ESI, Fig. S1 and S2†).

The hydroxide form of the salt was obtained by anion exchange of an aqueous solution with Ambersep 900(OH) ion exchange resin. The completion of the exchange was tested with a silver nitrate test and the concentration of hydroxide ions measured by titration with 0.1 M hydrochloric acid.

#### Synthesis of germanosilicate IM-12 (**UTL**)

The synthesis of zeolite IM-12 (**UTL**) was conducted according to literature protocols^58^,^59^ with a molar composition of 0.8 SiO_2_ : 0.4 GeO_2_ : 0.4 OSDA-OH : 35H_2_O. In a typical synthesis, 2.4 g Cab-O-Sil M5 (40 mmol) and 2.1 g germanium dioxide (20 mmol) were dissolved in 100 ml of a 0.6 M aqueous OSDA-solution and mechanically stirred for 30 min. The resulting gel was transferred into Teflon-lined stainless-steel autoclaves and heated at 175 °C for 7 days under static conditions. The product was recovered by filtration, thoroughly washed with water and dried overnight at 80 °C. Calcination was performed at 575 °C for 6 hours (with ramping speeds of 1 °C min^–1^ up and 2 °C min^–1^ down) in a tube furnace.

#### Ball milling-assisted hydrolysis of zeolite IM-12 (**UTL**)

Milling-assisted hydrolysis is conducted on a home-made rotary ball mill employing a 125 ml polypropylene bottle as the milling chamber. Prior to conducting the hydrolysis experiments, milling parameters were developed to achieve maximum efficiency. YTZ milling media (Tosoh, Japan) is employed for its high density and hardness. A media diameter of 3 mm was selected to maximise the number of collisions per unit time without overly compromising collision force. The optimum media : mill volume ratio and rotational speed were established with reference to the incline angle of the tumbling media and the critical speed (speed at which media centrifuge against the wall of the mill). 250 g of media and a rotation speed of 150 rpm (approximately 75% of the critical speed) were found to afford the maximum cascade length and maximum lift without incurring undue cataracting. 25 ml (enough to cover the media surface) of solvent (water or hydrochloric acid in various concentrations) was employed. 500 mg of calcined **UTL** were thus milled for varying time periods (typically 30 min). The resulting material was recovered with 50 ml ethanol, centrifugation and drying at 80 °C overnight. Calcinations were performed under the same conditions as above.

### Characterisations

#### PXRD

Powder X-ray diffraction patterns were collected on either PANalytical Empyrean diffractometer in reflection Bragg–Brentano mode or on a STOE STADIP diffractometer in transmission Debye–Scherrer mode. Both instruments are operated with monochromated Cu K_α1_ radiation.

#### SEM and EDS

Scanning Electron Microscopy images were obtained on a JEOL JSM-5600 using a thermionic tungsten filament or a JEOL JSM-6700F using a field emission gun. Both instruments are equipped with an Oxford INCA EDS system, used for elemental analysis.

#### TEM

Transmission Electron Microscopy images were obtained on a JEOL JEM NEOARM-200F, operated at 200 kV and collected with a TVIPS CMOS XF416 camera. The samples were prepared by a conventional dropping method, using acetone as dispersion medium and a holey carbon coated copper TEM-grid.

#### Solution-state NMR spectroscopy

Solution-state NMR spectra were obtained on a Bruker AV III 500, with a CryoProbe Prodigy BBO probe.

#### Solid-state NMR spectroscopy

^17^O and ^29^Si solid-state NMR spectra were collected on Bruker Avance III spectrometers, equipped with 14.1 T and 9.4 T wide-bore magnets, respectively, at Larmor frequencies of 81.4 MHz (^17^O) and 79.5 MHz (^29^Si) using a 4.0 mm low-*γ* HX probe. ^17^O spectra were acquired at 10 kHz MAS with a recycle interval of 1 s and are referenced to water (*δ*_iso_ = 0 ppm). A triple-quantum MAS NMR spectrum was acquired using a z-filtered pulse sequence^60^ and is shown after shearing (referenced in the indirect dimension using the convention by Pike *et al.*^61^). ^29^Si spectra were acquired at 14 kHz MAS with a recycle interval of 120 s and referenced to Q8M8 (octakis(trimethylsiloxy)silsesquioxane) (OSi(OMe)_3_) (*δ*_iso_ = 11.5 ppm).

## Results and discussion

The calcined samples of **UTL** were characterised as highly crystalline solids (using PXRD) with a Si/Ge ratio of 3.5–4 as determined by EDS. The crystals exhibit the typical rectangular plate habit with approximate dimensions of 35 μm × 45 μm × ∼1 μm.

**Fig. 3:**
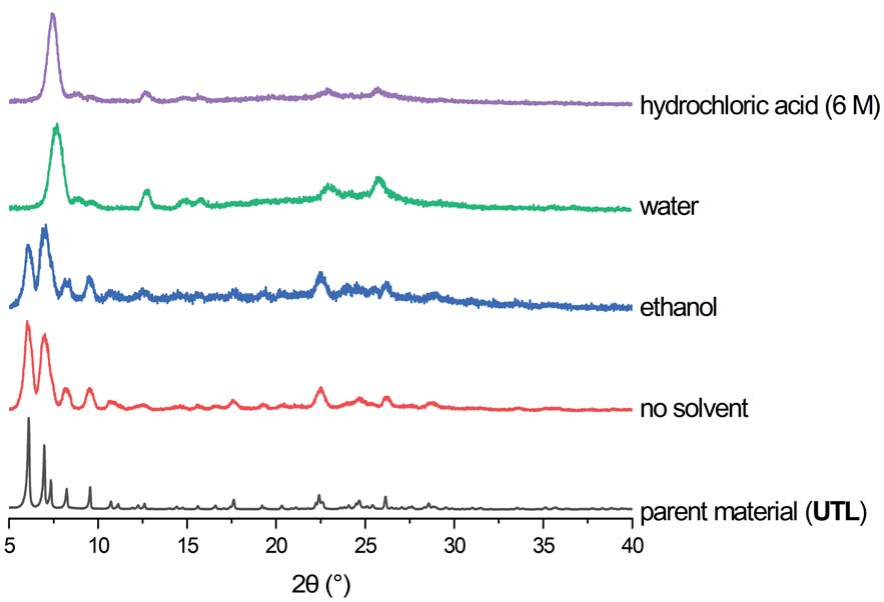
PXRD patterns of parent zeolite **UTL** and as made materials obtained from ball milling without solvent, and 25 ml of ethanol, water and 6 M hydrochloric acid.

**Fig. 4:**
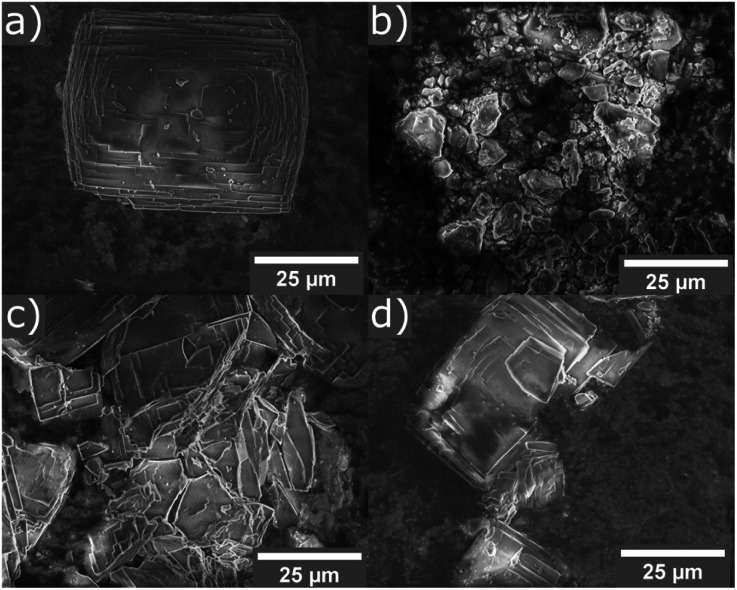
SEM images of parent zeolite **UTL** (a), and samples ball milled for 30 min without solvent (b), in 25 ml ethanol (c), and in 25 ml 6 M hydrochloric acid (d).

Hydrolysis experiments were performed in a home-made ball mill (see above) at room temperature and conducted for 30 minutes. A low liquid/solid ratio of 25 ml per 0.5 g of parent zeolite was chosen, which has been recently reported to yield good results at high temperatures.^46^ Several solvents were tested initially and their results showed the need for the presence of acid or water for successful hydrolysis, as neither solvent-free nor the use of “non-hydrolytic” solvents such as ethanol led to a breakdown of the **UTL** framework (see Fig. 3). The only discernible changes in the case of ethanol as solvent or an experiment without any liquid are reduced crystallinity and crystal size, as determined qualitatively from PXRD and SEM characterisation (Fig. 4). On the other hand, when using water or hydrochloric acid the parent zeolite was successfully hydrolysed. This means that the presence of an aqueous phase is required for the hydrolysis reaction to occur, in agreement with previous reports regarding the traditional ADOR process. The materials obtained through mechanochemically assisted disassembly consist of crushed pieces of the original sample, still clearly exhibiting some crystalline character but reduced order (PXRD, Fig. 3 and SEM, Fig. 4).

This result shows notable improvements regarding the reaction conditions compared to the conventional ADOR process. The decreased amount of liquid medium required means a reduced amount of waste is produced. Similarly, energy resources are used much more economically in the mechanochemical approach. The reaction time was shortened considerably, to only half an hour, a significant improvement over the conventional overnight reaction. Additionally, avoiding an external heating source and performing the hydrolysis at room temperature helps keeping the overall energy consumption low. This is an additional effect of performing the reaction in a mechanochemically assisted manner, since despite the bulk temperature not significantly increasing beyond ambient conditions, the local temperature can rise due to the exerted frictional and mechanical forces of the milling process.

Based on these preliminary results, the following studies are focused on investigation of acidity and reaction time on the zeolite transformation.

### Hydrolysis with hydrochloric acid

In a detailed study, Wheatley *et al.*^59^ showed that the concentration of hydrochloric acid used in an ADOR reaction has a profound influence on the structure of the resulting product. Tuning of the pore sizes in the final product can be achieved by carefully selecting the acidity of the liquid medium. The previously known IPC-2 (**UTL**-s4r: *i.e.* the parent **UTL** zeolite but with loss of a s4r unit between the layers) and IPC-4 (**UTL**-d4r), can be prepared in this way. In addition, the intermediate frameworks IPC-6 (combination of **PCR** and **OKO** connections, recently assigned the framework code *PCS by the IZA^3^,^20^) and IPC-7 (combination of **OKO** and **UTL** connections) are accessible using this approach. In this vein, a study of the disassembly process using hydrochloric acid in varying concentrations was conducted in the ball mill.

**Fig. 5:**
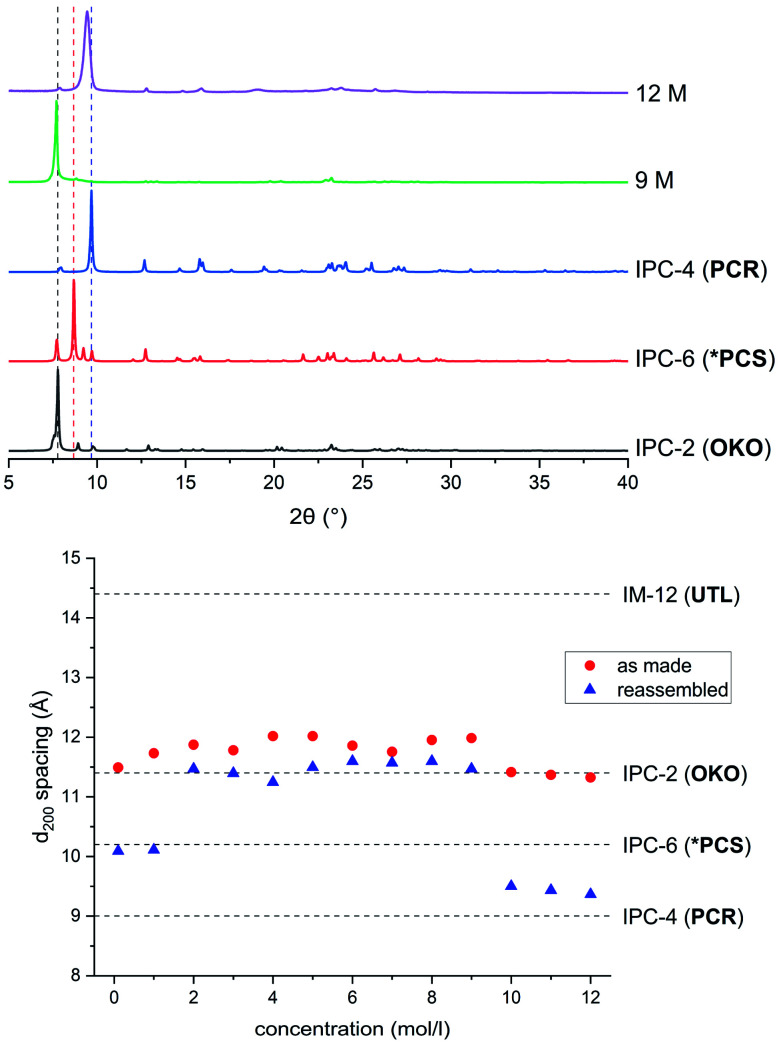
Top: PXRD patterns of samples hydrolysed with 9 M and 12 M HCl and subsequently reassembled, compared to ideal IPC-2, IPC-6 and IPC-4. Bottom: *d*_200_ spacings for as made (red, filled circles) and reassembled (blue, triangles) samples ball milled with HCl in concentrations varying from 0.1 M to 12 M.

The products of the hydrolysis of the parent zeolite **UTL** can be described as IPC-6P- or IPC-2P-like materials, as both these materials exhibit similar PXRD patterns (see ESI, Fig. S3†). These intermediate materials are disordered precursors of their respective more ordered fully connected end-products (IPC-2, ICP-6). Lower *d*_200_ values indicate an IPC-6P framework, whereas larger distances hint at the formation of IPC-2P (compare Fig. 2). There is no straightforward dependency on acid concentration of the products formed from the mechanochemical experiments shown in Fig. 5. The *d*-spacing corresponding to the main peak (the 200 reflection) initially increases with acid concentration, stabilises at intermediate molarities and decreases again for concentrated HCl solutions. This is different to the results in the traditional ADOR process where the concentrated HCl solutions give only IPC-2P. This difference may be related to the solubility of the germanium species after hydrolysis. In the presence of hydrochloric acid, the germanium-containing d4r unit is attacked, forming chlorinated germanium species which then further hydrolyse to GeO_2_.^48^ The concentration of both chloride and hydronium ions (protons) work in tandem in this disassembly of the zeolite layers; low pH values facilitate bond breaking and the presence of chloride ions ensures a driving force towards product (hydrolysed Ge species) formation. In the traditional synthesis there is enough solvent to ‘wash out’ the Ge-containing species from between the layers but there may not be enough liquid for the same thing to happen in the mechanochemical approach. The solubility of GeO_2_ reaches a minimum at 5 M hydrochloric acid,^62^ which coincides with the maximum reached for the *d*_200_-spacing of the produced materials.

Support for this reasoning comes from the PXRD measurements of the ball milled ADOR products (see ESI, Fig. S3†) which show a minor peak at 26° 2*θ* from α-Quartz type GeO_2_. This germanium species has recently been shown to be recyclable in an ensuing synthesis of parent germanosilicate zeolite when high amounts of liquid are used.^50^ The low amount of solvent used in this study is a likely cause for the insufficient removal of the germanium containing by-product. The residual Ge species causes a higher average interlayer distance than would be expected, thus the larger measured *d*_200_ spacing. Additionally, EDS analyses reveals a higher germanium content for ball-milled samples compared to those formed in conventional round-bottom flask hydrolysis, where the products are essentially purely siliceous zeolite phases due to complete removal of the germanium species in the larger volume of solvent present. The Si/Ge ratio of products from the ball milling reaction increases with HCl concentration from 10 in dilute acid solutions up to 30 for concentrated hydrochloric acid.

Reassembly of the products through heat treatment is necessary to further elucidate their structure (see ESI, Fig. S4† for all PXRD patterns). Condensation of IPC-2P to IPC-2 would only show a negligible change to the position of the (200) reflection and therefore its interlayer spacing, whereas the difference between IPC-6 and its precursor is known to be 1.3° 2*θ* (a difference in *d* of ∼1 Å).^20^ The samples obtained from hydrolysis with 0.1 M and 1 M HCl reconnected to form IPC-6 type frameworks. Increasing the acidity led to mixtures of IPC-6 and IPC-2 structures, where seemingly s4r units are again formed in the interlayer region and the character of the final framework is closer to **OKO**. Upon reaching a concentration of 9 M HCl, the product exhibits a well-ordered IPC-2 structure. Despite the above mentioned higher solubility of the disassembly by-product, it appears that the increased rate of Si–O (and possibly to a minor degree Ge–O) bond formation at lower pH^63^ stabilises the IPC-2P precursor, which is then transformed into the final product with the **OKO** framework. Up to concentrations of 9 M HCl, the results are as one would expect from comparison with the traditional ADOR approach.

However, the PXRD patterns of samples produced from highly acidic reactions ([H^+^] = 10 M to 12 M) display a shift of the main peak (corresponding to the (200) reflection) to higher 2*θ* angles. Using concentrated HCl, the diffraction pattern collected is strikingly similar to IPC-4 after calcination. The *d*_200_ peak in the PXRD pattern is not at exactly the same 2*θ* value as one would expect from the ideal framework, with a difference of *circa* 0.2°, indicating the presence of some disorder due to a small amount of remaining s4r linkages (approximately 14% of interlayer linkages judging from the peak position), which leads to an increased average interlayer spacing and correspondingly a shift to a lower 2*θ* angle.

Through the conventional ADOR route, zeolite IPC-4 is obtained exclusively by thorough and complete hydrolysis of parent **UTL** into its layered form IPC-1P, subsequent organisation *via* intercalation using octylamine and final condensation. The direct accessibility of a zeolite with the **PCR** framework using ball milling, combining disassembly and organisation in one step, is a considerable improvement from an economical point of view.

These results show that there is a considerable difference between samples obtained through previously published preparation techniques and the method showcased in this study. This is most prominent in the samples obtained at acid concentrations from 9 M and 12 M HCl, which were therefore subjected to additional investigations.

Nitrogen adsorption experiments for the two selected samples were conducted to compare them to reported zeolites with **OKO** and **PCR** frameworks. The calculated BET surface area for the sample with an IPC-2 type structure was 368 m^2^ g^–1^, which is in very good agreement with literature values.^13^ The IPC-4-like material exhibits only 60 m^2^ g^–1^, significantly lower then expected values, which are typically in the range of 200–300 m^2^ g^–1^.^13^ The suggested disorder in the structure of the IPC-4 material from PXRD data probably provides the reason as interruptions in the layer stacking lead to blockages of pore channels, thus lowering the measured BET area.

**Fig. 6:**
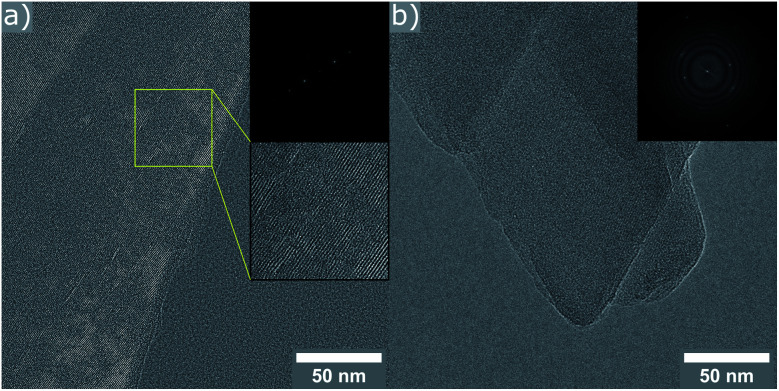
HR-TEM images with FFT inserts of the sample prepared with 12 M HCl. (a) Crystal exhibiting lattice fringes with a distance of 9.3 Å (indicative of **PCR** type framework), as well as defective regions (highlighted area). (b) Crystals of different orientation with *d*-spacings corresponding to (200), (020), and (420) of the **PCR** framework.

In an effort to confirm these conclusions, high-resolution TEM images were collected for the two reassembled samples. In the case of the product obtained with 9 M HCl, only crystals exhibiting a *d*-spacing corresponding to an **OKO** linkage were found (11.2 Å). The material resulting from the experiment using 12 M acid, consisted of crystals showing lattice fringes, whose spacing is in very good agreement with the results from PXRD (9.4 Å for *d*_200_). Some particles showed regions with a slightly increased *d*-spacing (see ESI, Fig. S12†), in line with the explanation of disorder above. Further investigation of the crystals with the **PCR**-like connectivity reveal interruptions in the structure (marked area in Fig. 6). These give a good explanation for the low value measured for the BET surface area, as the pore channel connectivity is no longer reaching throughout the entire crystal.

A study investigating the influence of reaction time on products using both 9 and 12 M HCl concentrations was also conducted. Only a minor change giving a marginally increased interlayer spacing can be observed over the first 2 hours, stabilising thereafter (see ESI, Fig. S5–S8†). The reduced particle size due to the exerted mechanical forces is thought to be one of the main causes for the decreased reaction time. The shorter diffusion paths for the hydrolysis reagent required to reach the labile parts of the framework are a likely reason for this observation. Another factor may also be the increased local temperature caused by the milling. Previous work using conventional hydrolysis showed the preferential formation of IPC-2-like materials for prolonged reaction times due to rearrangement of the layered intermediate species.^18^,^59^ In contrast, the ball milling procedure does not follow this observation and the interlayer spacing of the frameworks produced remains the same throughout the investigated timeframe. The usual additional intercalation of siliceous s4r units within the interlayer spaces, fed by silicon from the layers themselves, is inhibited in the ball milling assisted hydrolysis.

### Hydrolysis with water

**Fig. 7:**
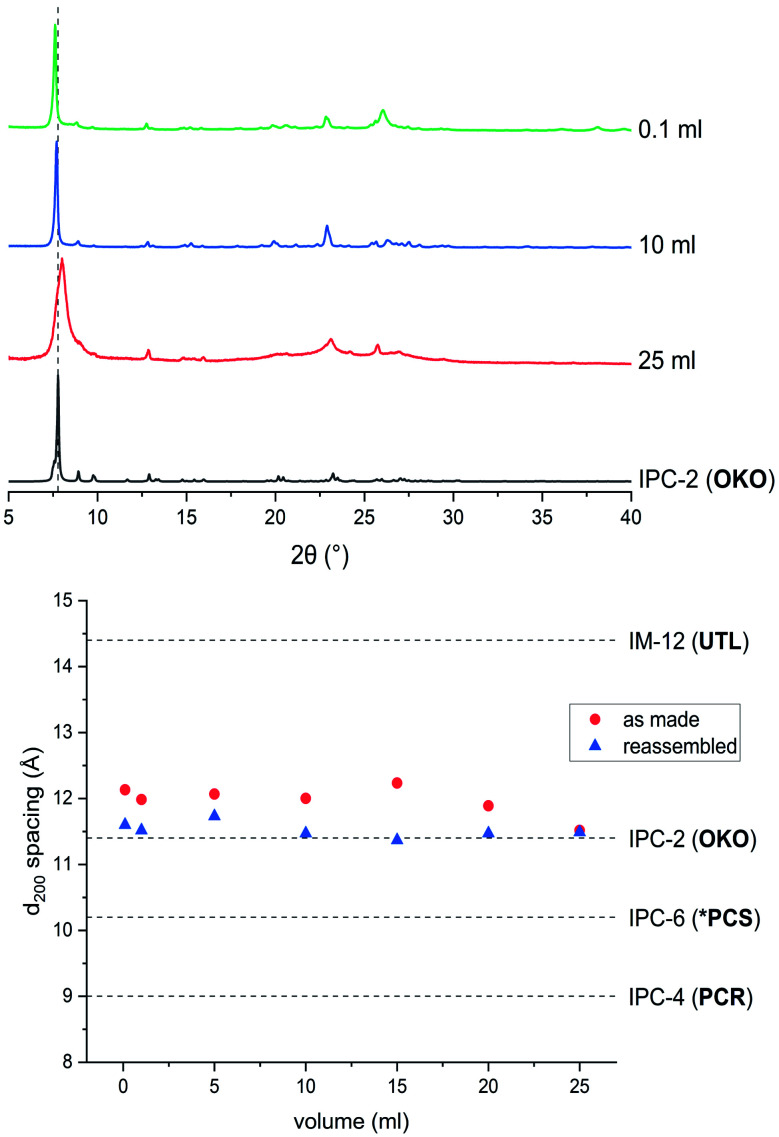
PXRD patterns of as made (top) and calcined (bottom) materials, obtained from ball milling experiments with varying amounts solvent (water). Reaction time was 30 min for all samples.

Hydrolysis of **UTL** is not restricted to acidic media but also occurs when using water as reagent. Judging from PXRD data (Fig. 3), the structure of as made hydrolysed material from the reaction with water is of similar nature to the products found when hydrolysing with acid, exhibiting patterns of corresponding to IPC-2P/IPC-6P structures (see ESI, Fig. S9†). In order to determine whether reassembly would lead to different materials than samples treated with dilute acid (*i.e.* similar pH), reassembly was performed. Reactions with 0.1 and 1 M HCl lead to materials exhibiting IPC-6 frameworks, whereas with no acid present, the structure of the product is much closer to IPC-2 as shown in Fig. 7 (and ESI, Fig. S10†). TEM analysis confirms the PXRD result, revealing lattice fringes with a spacing of 11.3 Å (see ESI, Fig. S10†).

An explanation for this behaviour may be the slower hydrolysis kinetics when using water as the reagent compared to hydrochloric acid, even in dilute concentration.^23^,^63^ The disassembly is controlled not only by the pH of the liquid phase, but is also influenced by the presence of chloride ions. Removing the ability to form chlorinated germanium species during hydrolysis means that disassembly is not progressing as fast, therefore stopping at the stage of IPC-2P and the resulting IPC-2 structure upon condensation.

### Low volume hydrolysis and ^17^O enrichment for solid-state NMR spectroscopy

Since the hydrolysis reaction with 25 ml of water was successful, reduction of the solvent volume used was undertaken. Fig. 7 shows the dependency of the resulting structures (both as made and calcined materials) as the volume decreases from 25 ml to 0.1 ml. This translates to a parameter *η* of 0.2; a typical value for liquid assisted grinding (LAG)^64^,^65^ and a similar ratio as used by the Laurencin group in Montpellier for their enrichment studies on metal oxides like CaO.^55^,^56^ Even with the low, reagent-level amount of water, the zeolite is successfully disassembled, and subsequently reassembled to an IPC-2-like material. As is common in mechanochemical treatments, the particle morphology deteriorates significantly, with comparatively low crystallinity and broader peaks in PXRD patterns and diminished crystallite size and agglomeration visible in SEM images (see ESI, Fig. S11†). This result is not surprising due to the increased contact of milling media with the zeolite sample and the lack of excess liquid to dampen the mechanical force and subsequent amorphization. The effect of mechanochemical treatment on the material is evident in the ^29^Si NMR spectrum of the hydrolysed product (see ESI, Fig. S13†). Although the diffraction-derived hydrolysis product is in line with that expected for the solid : solvent ratio used,^51^ the *Q*^3^ : *Q*^4^ ratio determined from analytical fitting is 1 : 3.1; far closer to highly disordered IPC-1P (1 : 2.5) than that for IPC-2P (1 : 7). The greater number of silanols generated and incomplete removal of interlayer silicon species observed by NMR spectroscopy can also be attributed to the increased amorphization effect of the ball milling treatment and low levels of washing liquid.

A significant consequence of hydrolysis in very low volumes of water is the possibility to use this methodology to enrich samples with the NMR-active isotope of oxygen, ^17^O. The ability to obtain ^17^O NMR data is highly desired in the study of reactive microporous materials.^51^ However, the routine study of ^17^O by NMR spectroscopy is hindered by its low natural abundance (0.037%), quadrupolar spin (*I* = 5/2) and moderate gyromagnetic ratio, and acquisition of spectra could take weeks or even months to obtain a sufficient signal-to-noise ratio. Hence, isotopic enrichment using a limited range of expensive commercially available reagents (H_2_^17^O_(l)_, ^17^O_2(g)_) is usually required.^66^,^67^ In order to ensure enrichment is financially feasible, procedures have to be optimised to work with very low amounts of the enriched reagent, which is where the low volume reaction comes into play. In this work, 100 μL of 40% ^17^O enriched water (600 € per ml) was used to produce a sample with a higher amount of the NMR active nucleus.

**Fig. 8:**
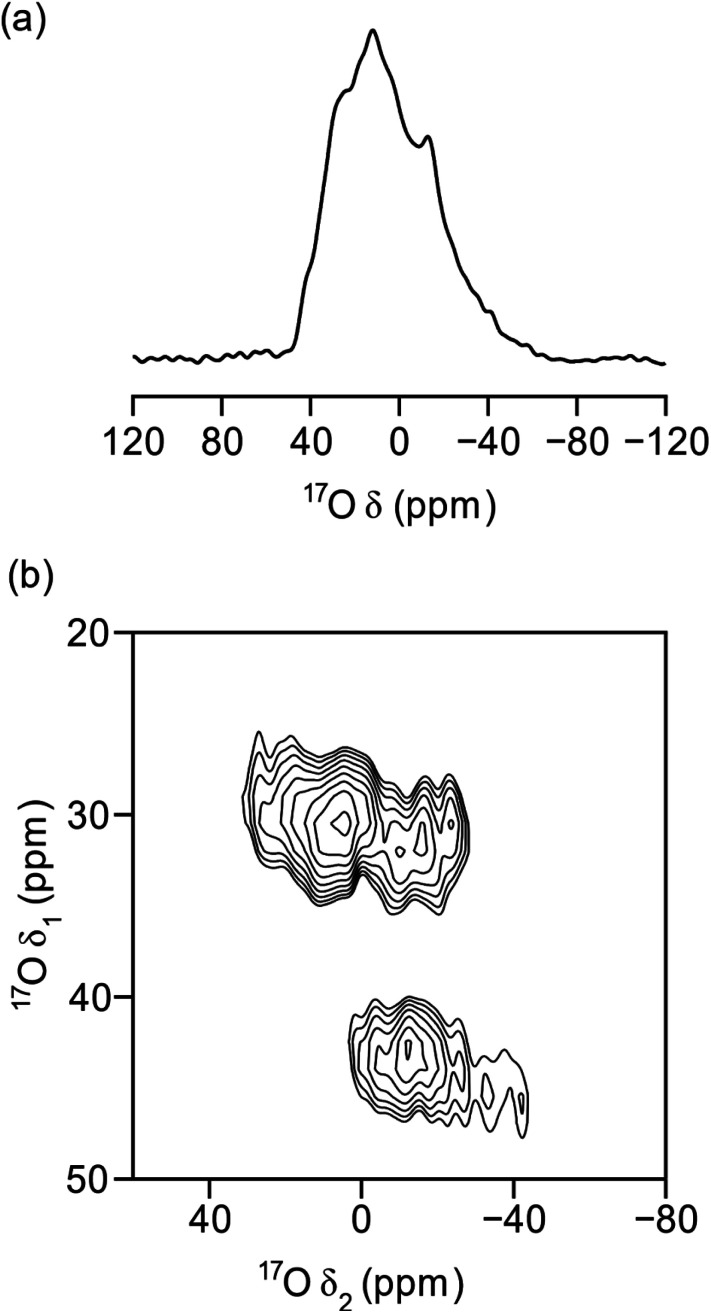
^17^O (14.1 T, 10 kHz) ^1^H-decoupled (a) MAS and (b) MQMAS NMR spectra of calcined **UTL**, ball milled in 40% H_2_^17^O for 30 minutes and dehydrated under vacuum at 120 °C overnight. The spectrum in (b) is shown after a shearing transformation.

Fig. 8 demonstrates the successful enrichment of the **UTL** framework when ball-milled with 100 μL of 40% H_2_^17^O at room temperature. The ^17^O magic angle spinning (MAS) NMR spectrum, acquired with a spin-echo,^68^ (Fig. 8a) shows a broad overlapped signal between a *δ* = –40–40 ppm, as a result of the quadrupolar broadening. The ^1^H-decoupled ^17^O multiple-quantum (MQ) MAS^60^,^69^ spectrum in Fig. 8b shows three separate signals. One of these can be attributed to Si-^17^O–Si groups (*δ*_1_, *δ*_2_ = 30,0–30 ppm)^51^,^70^–^72^ and a second to crystalline quartz-phase GeO_2_ (*δ*_1_ = 45 ppm). The identity of the latter signal was achieved by adaption of a previously published procedure for the synthesis of Ge^17^O_2_, and subsequent NMR analysis of the product.^73^ This assignment verifies the interpretation of the PXRD patterns above. The third environment resolved by MQMAS at (*δ*_1_, *δ*_2_ = 28,–30–0 ppm) is yet to be unambiguously assigned; however, it is thought that this resonance may be related to Si-^17^O–Ge formed in the milling process. Comparing the experimental time required to acquire the spectra in Fig. 8a and b (2 h and 16 h, respectively) to those required in previous work for ^17^O-enriched pyrochlores,^74^ zeolites^75^,^76^ and MOFs,^54^ with enrichment levels (as determined by mass spectrometry) of 5–10%, 15–25% an 15–20%, respectively, suggests the levels of enrichment observed in the ball-milled ADOR products are approximately 10%. This is in excellent agreement with the maximum level of enrichment (calculated from the levels of ^16^O and ^17^O in all reagents) of ∼11%.

## Conclusions

Using a ball mill for mechanochemically assisted hydrolysis of germanosilicate zeolite **UTL** has been successful. Improvements regarding reaction time, lowering of reaction temperature and of solvent volume, relative to conventional ADOR have been achieved. The materials obtained are known hydrolysis products of **UTL**, albeit with a higher degree of disorder compared to conventional syntheses, likely caused by the ball milling procedure itself. Using 12 M hydrochloric acid during milling, a product with high structural similarity to the **PCR** framework has been obtained directly, without the commonly required complete disassembly to layered precursor IPC-1P and the subsequent organisation step including an organic additive.

Three factors can be determined to cause the disparities between previously published reports and the data presented: (1) the short reaction time of 30 min, (2) the low temperature, since no heating source was employed, and (3) the low liquid/zeolite ratio of 25 ml/0.5 g. The combination of these leads to ICP-2 (**OKO**), IPC-6 (*PCS) with varying degrees of disorder and amorphization, and IPC-4 (**PCR**) type materials, depending on the concentration of used hydrochloric acid or the usage of water.

Additionally, the developed methodology has shown that reagent-levels (*η* = 0.2) of water are sufficient to induce the disassembly process of the parent germanosilicate. This can be exploited for cost-efficient ^17^O enrichment of the zeolite products and intermediates providing the additional opportunity for solid-state NMR spectroscopic characterisation of these materials, at very little financial cost and with high atom efficiency. The procedure presented here is thought to be applicable to any ADORable zeolite.

The mechanochemically assisted synthesis of ADOR zeolites presented here demonstrates how employing mechanical force can result in savings in energy, time and reagent costs in the synthesis and isotopic enrichment of hydrothermally inaccessible zeolites.

## Author contributions

R. E. M. designed the project. D. N. R. performed synthesis of materials, PXRD and electron microscopy and analysis of the results. S. J. W. developed the protocol for ball milling. C. M. R. and S. E. A. designed and performed solid-state NMR spectroscopic measurements and analysed the results. D. N. R. and R. E. M. wrote the paper. All authors discussed the results and edited the manuscript.

## Data access

The research data underpinning this publication can be accessed at https://doi.org/10.17630/6c2e36ec-193c-4588-91dc-620500b56d80.

## Conflicts of interest

There are no conflicts of interest to declare.

## Supplementary Material

Supplementary information
