# Supplementary material for: Mechanochemically assisted hydrolysis in the ADOR process
Source: Chem Sci. 2020 Jun 15;11(27):7060–9. doi: 10.1039/d0sc02547j (PMC7499815; doi:10.1039/d0sc02547j)
Supplement: Supplementary file 1 [file SC-011-D0SC02547J-s001.pdf]

Electronic Supplementary Material (ESI) for Chemical Science.
This journal is © The Royal Society of Chemistry 2020

Electronic Supporting Information

Mechanochemically assisted hydrolysis in the ADOR process

Daniel N. Rainer[a], Cameron M. Rice[a], Stewart, J. Warrender,[a] Sharon E. Ashbrook[a] and Russell E. Morris[a,b,*]

[a]School of Chemistry, EaStCHEM, University of St. Andrews, North Haugh, St. Andrews, Fife, KY16 9ST, UK.
[b]Department of Physical and Macromolecular Chemistry, Faculty of Sciences, Charles University, Hlavova 8, 128 43 Prague 2, Czech Republic.

Figure S1: ^{1}H NMR of the OSDA (6R,10S)-6,10-dimethyl-5-azoniaspiro[4.5]decane bromide. 2
Figure S2: ^{13}C NMR of the OSDA (6R,10S)-6,10-dimethyl-5-azoniaspiro[4.5]decane bromide. 2
Figure S3: PXRD patterns of as made materials produced from ball milling UTL in varying concentrations of hydrochloric acid. 3
Figure S4: PXRD patterns of reassembled materials produced from ball milling UTL in varying concentrations of hydrochloric acid. 3
Figure 5: PXRD patterns of as made materials produced from ball milling UTL in 9 M hydrochloric acid for different times. 4
Figure 6: PXRD patterns of reassembled materials produced from ball milling UTL in 9 M hydrochloric acid for different times. 4
Figure S7: PXRD patterns of as made materials produced from ball milling UTL in 12 M hydrochloric acid for different times. 5
Figure S8: PXRD patterns of reassembled materials produced from ball milling UTL in 12 M hydrochloric acid for different times. 5
Figure S9: PXRD patterns of as made materials produced from ball milling UTL with different volumes of water. 6
Figure S10: PXRD patterns of reassembled materials produced from ball milling UTL with different volumes of water. 6
Figure S11: SEM images of various ball milled and reassembled samples. 7
Figure S12: TEM images with FFT inserts. 8
Figure 13: ^{29}Si (9.4 T, 14 kHz) MAS NMR spectrum of calcined UTL, ball milled in 40% $H_2{}^{17}O$ for 30 minutes and dehydrated under vacuum at 120 ℃ overnight. 9

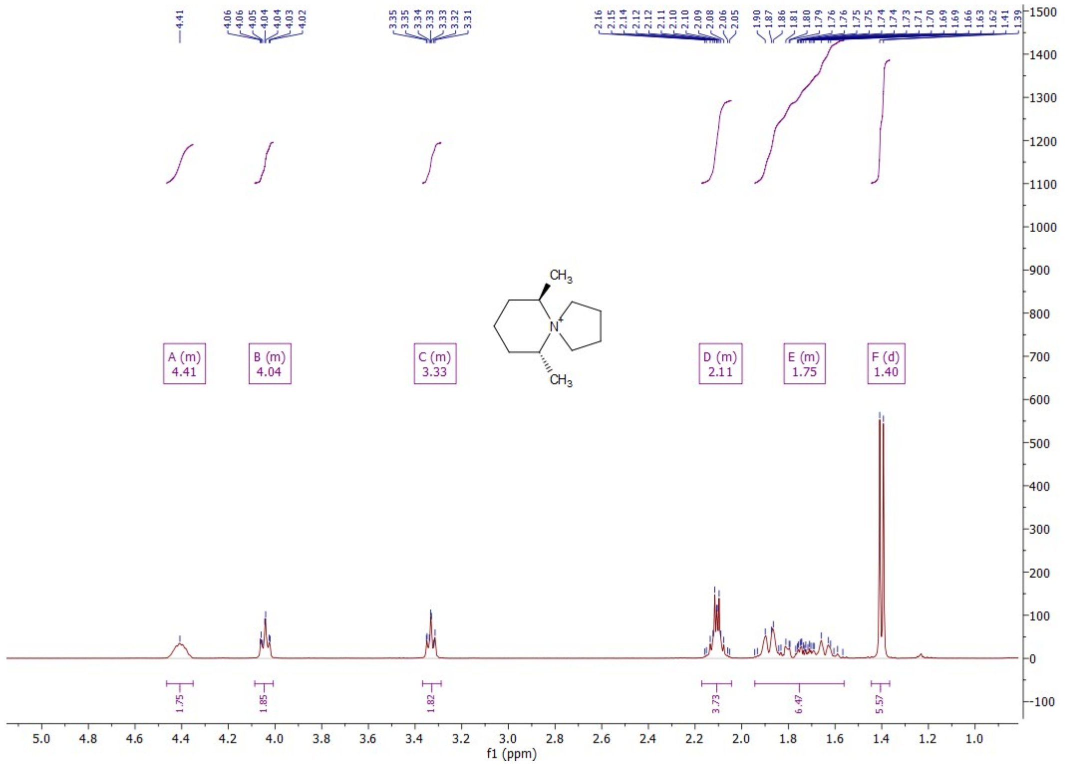

Figure S1: ^{1}H NMR of the OSDA (6R,10S)-6,10-dimethyl-5-azoniaspiro[4.5]decane bromide.

^{1}H NMR (400 MHz, Chloroform-d) δ 4.46–4.35 (m, 2H, NC$\underline{H}$), 4.09–4.01 (m, 2H, NC$\underline{H_2}$), 3.37–3.29 (m, 2H, NC$\underline{H_2}$), 2.17–2.04 (m, 4H, NCH$_2$C$\underline{H_2}$), 1.94–1.56 (m, 6H, C$\underline{H_2}$C$\underline{H_2}$C$\underline{H_2}$CHN), 1.40 (d, J = 6.5 Hz, 6H, C$\underline{H_3}$).

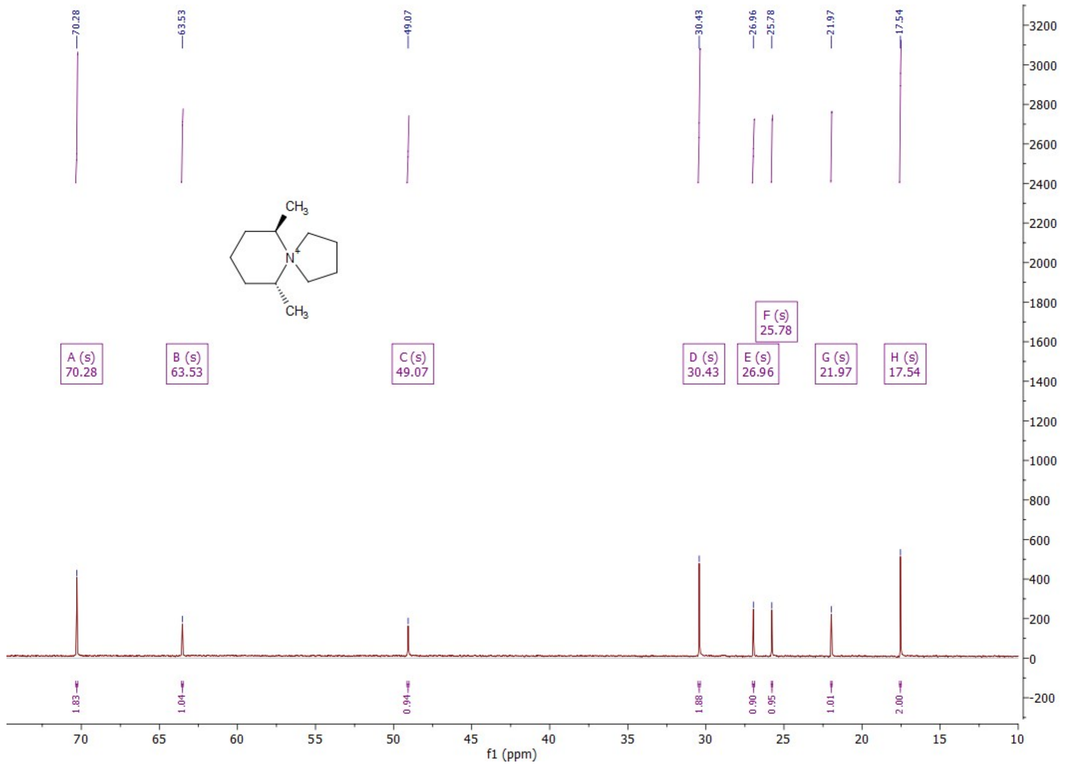

Figure S2: ^{13}C NMR of the OSDA (6R,10S)-6,10-dimethyl-5-azoniaspiro[4.5]decane bromide.

^{13}C NMR (126 MHz, Chloroform-*d*) δ 70.28, 63.53, 49.07, 30.43, 26.96, 25.78, 21.97, 17.54.

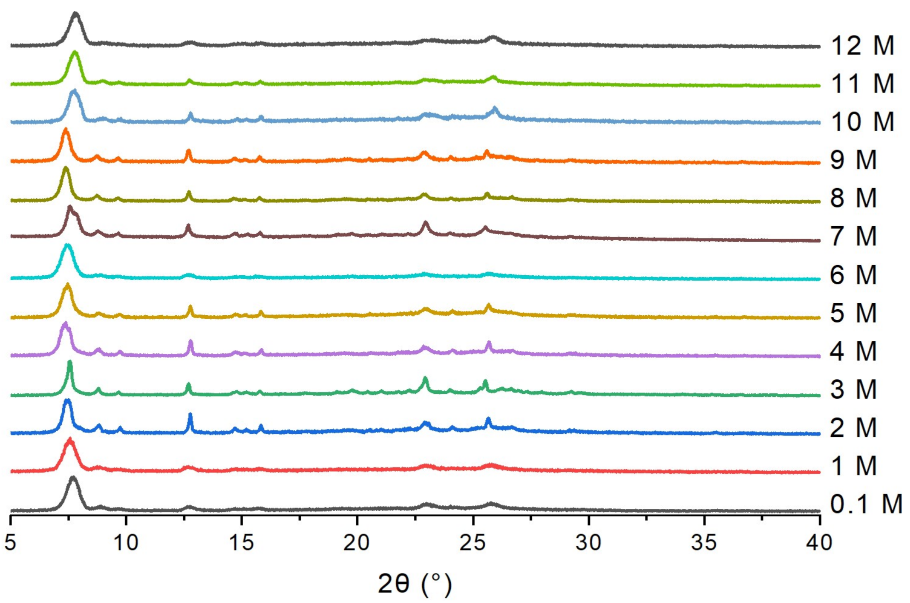

Figure S3: PXRD patterns of as made materials produced from ball milling UTL in varying concentrations of hydrochloric acid.

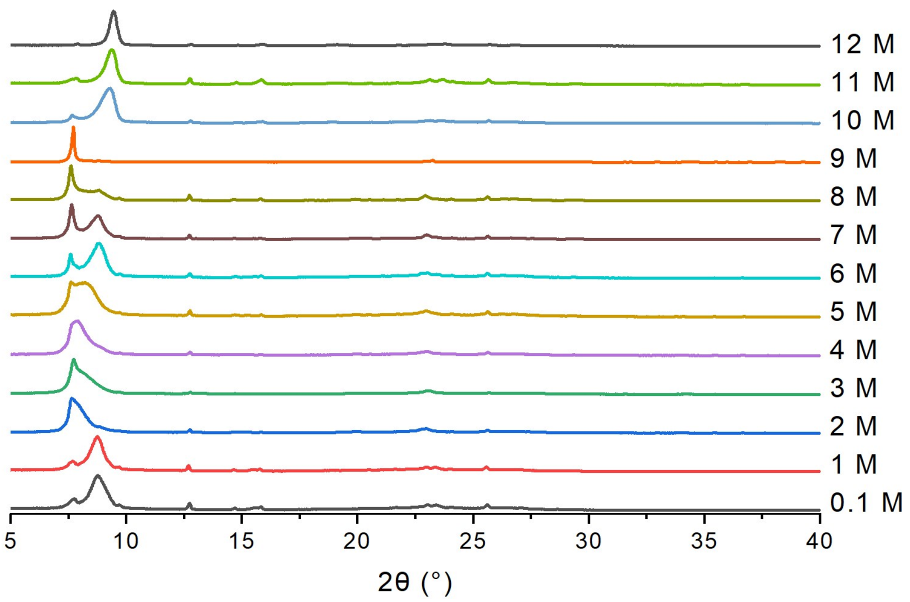

Figure S4: PXRD patterns of reassembled materials produced from ball milling UTL in varying concentrations of hydrochloric acid.

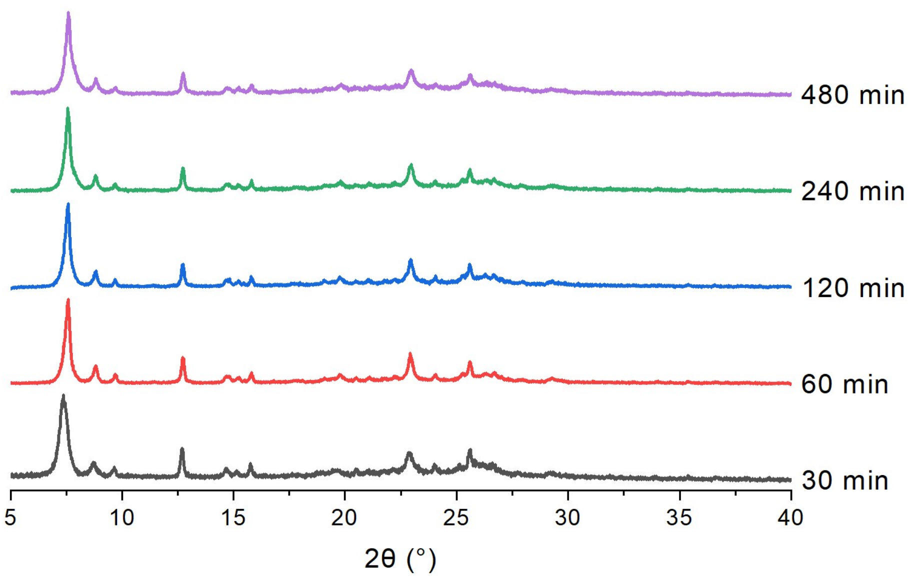

Figure 5: PXRD patterns of as made materials produced from ball milling UTL in 9 M hydrochloric acid for different times.

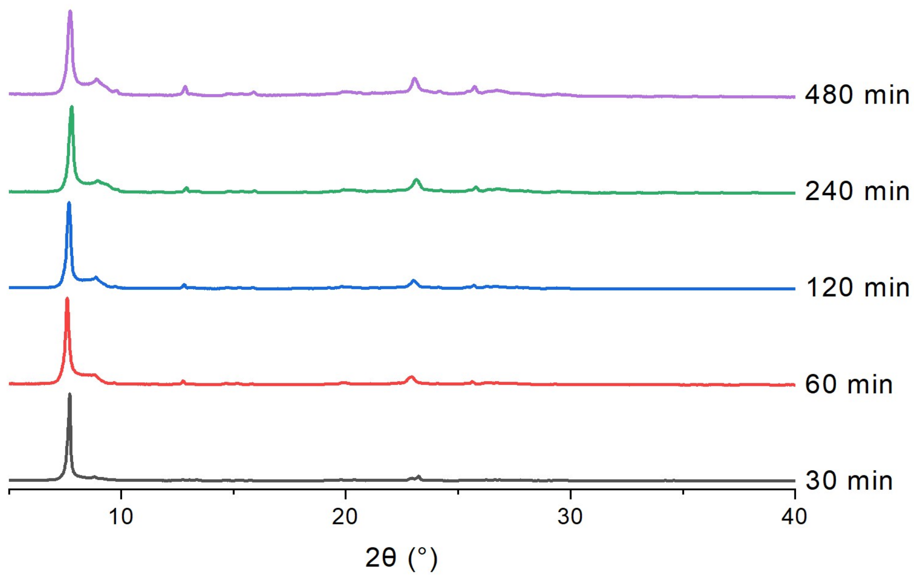

Figure 6: PXRD patterns of reassembled materials produced from ball milling UTL in 9 M hydrochloric acid for different times.

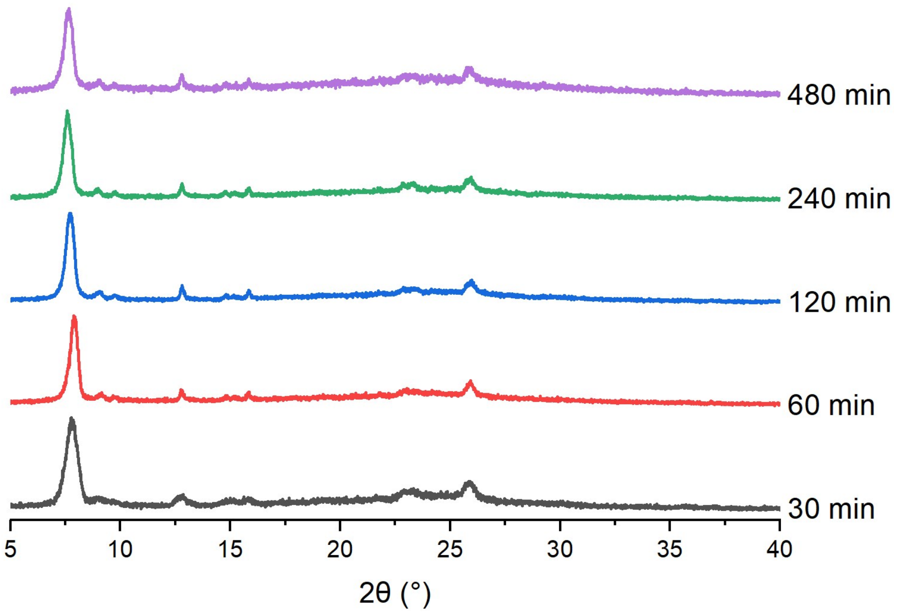

Figure S7: PXRD patterns of as made materials produced from ball milling UTL in 12 M hydrochloric acid for different times.

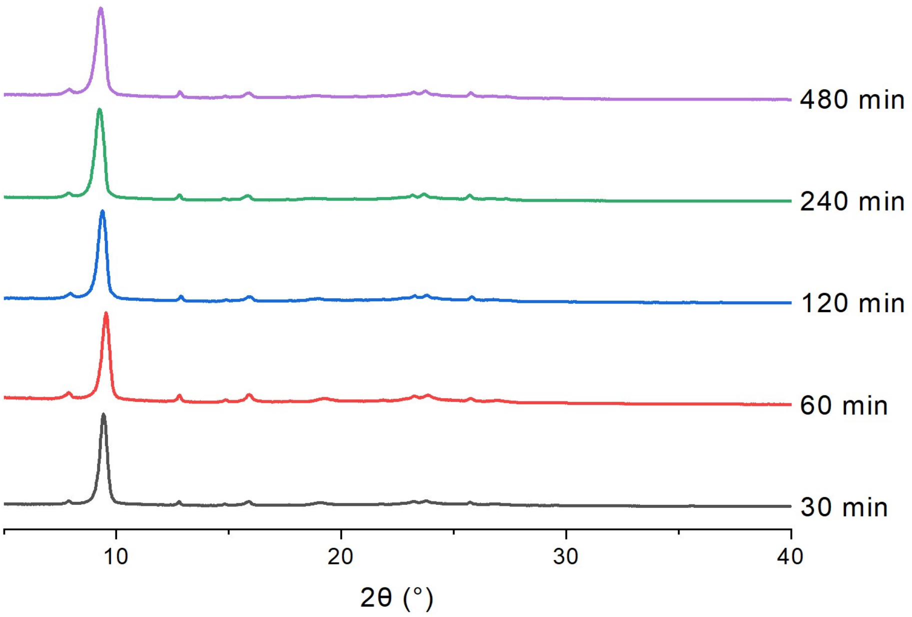

Figure S8: PXRD patterns of reassembled materials produced from ball milling UTL in 12 M hydrochloric acid for different times.

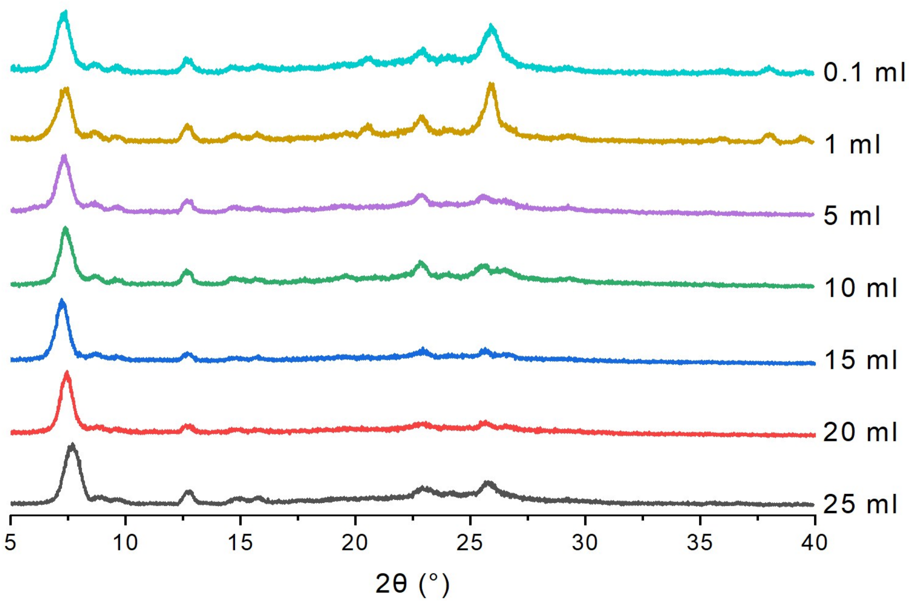

Figure S9: PXRD patterns of as made materials produced from ball milling UTL with different volumes of water.

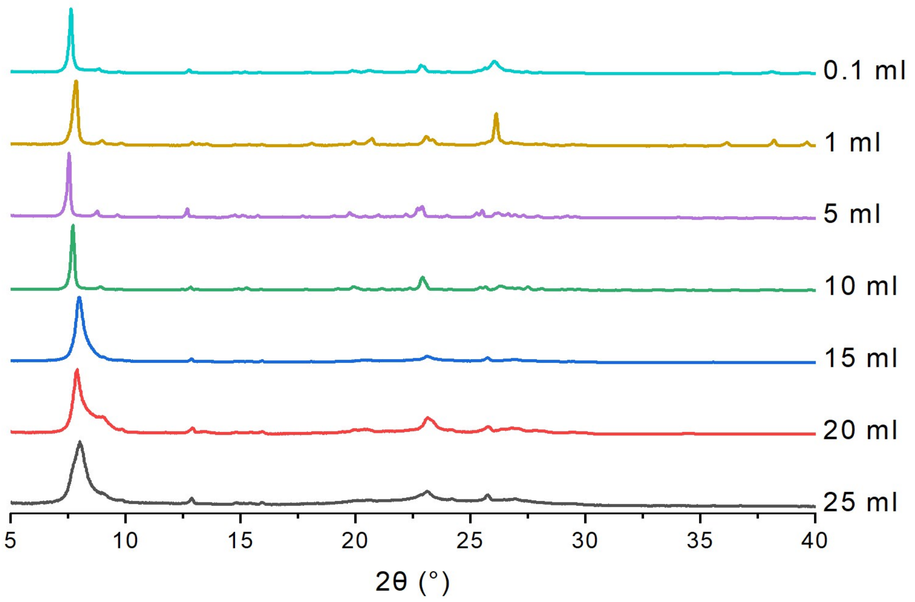

Figure S10: PXRD patterns of reassembled materials produced from ball milling UTL with different volumes of water.

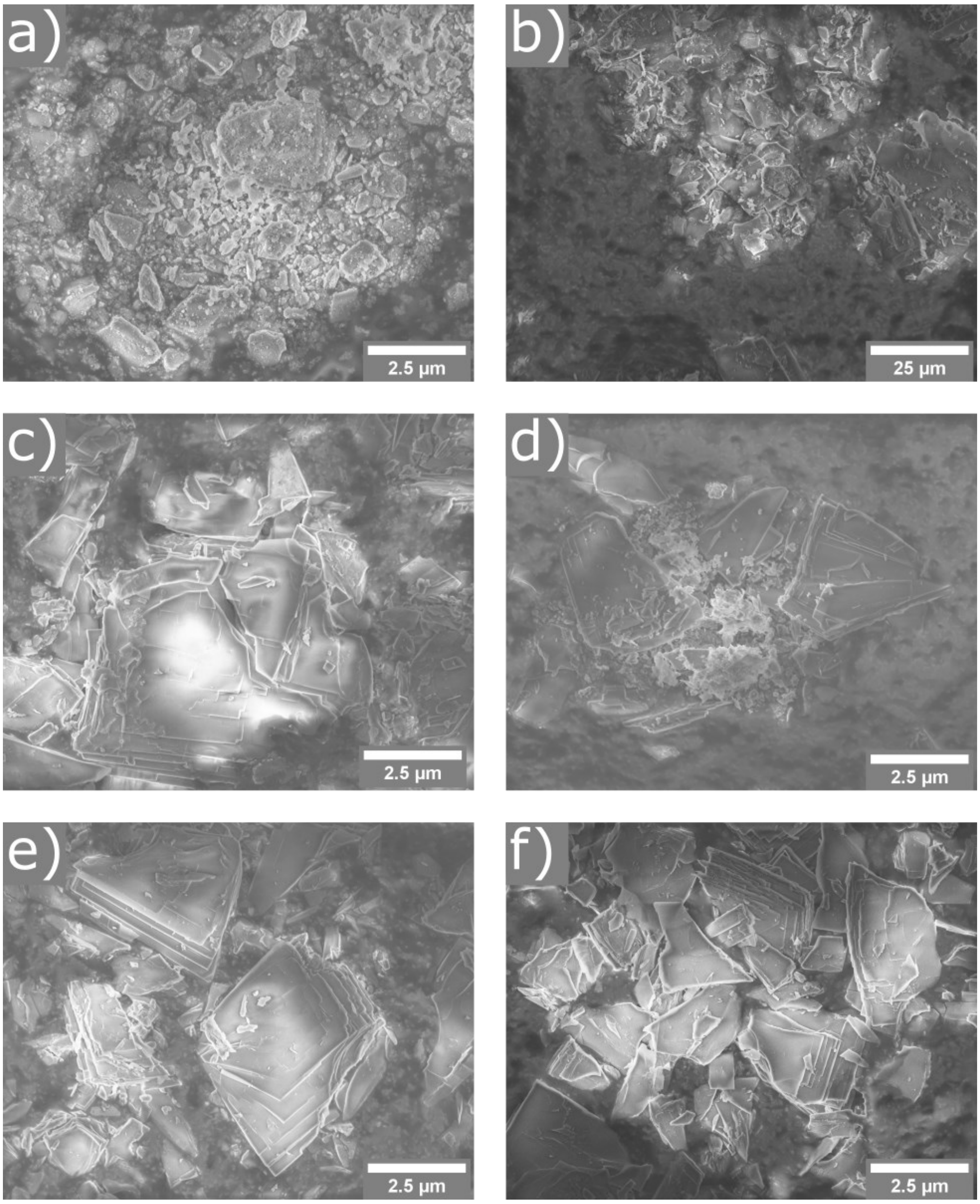

Figure S11: SEM images of various ball milled and reassembled samples.

a) 25 ml water; b) 5 ml water; c) 0.1 ml water; d) 6 M HCl; e) 9 M HCl; f) 12 M HCl.

Figure S12: TEM images with FFT inserts.

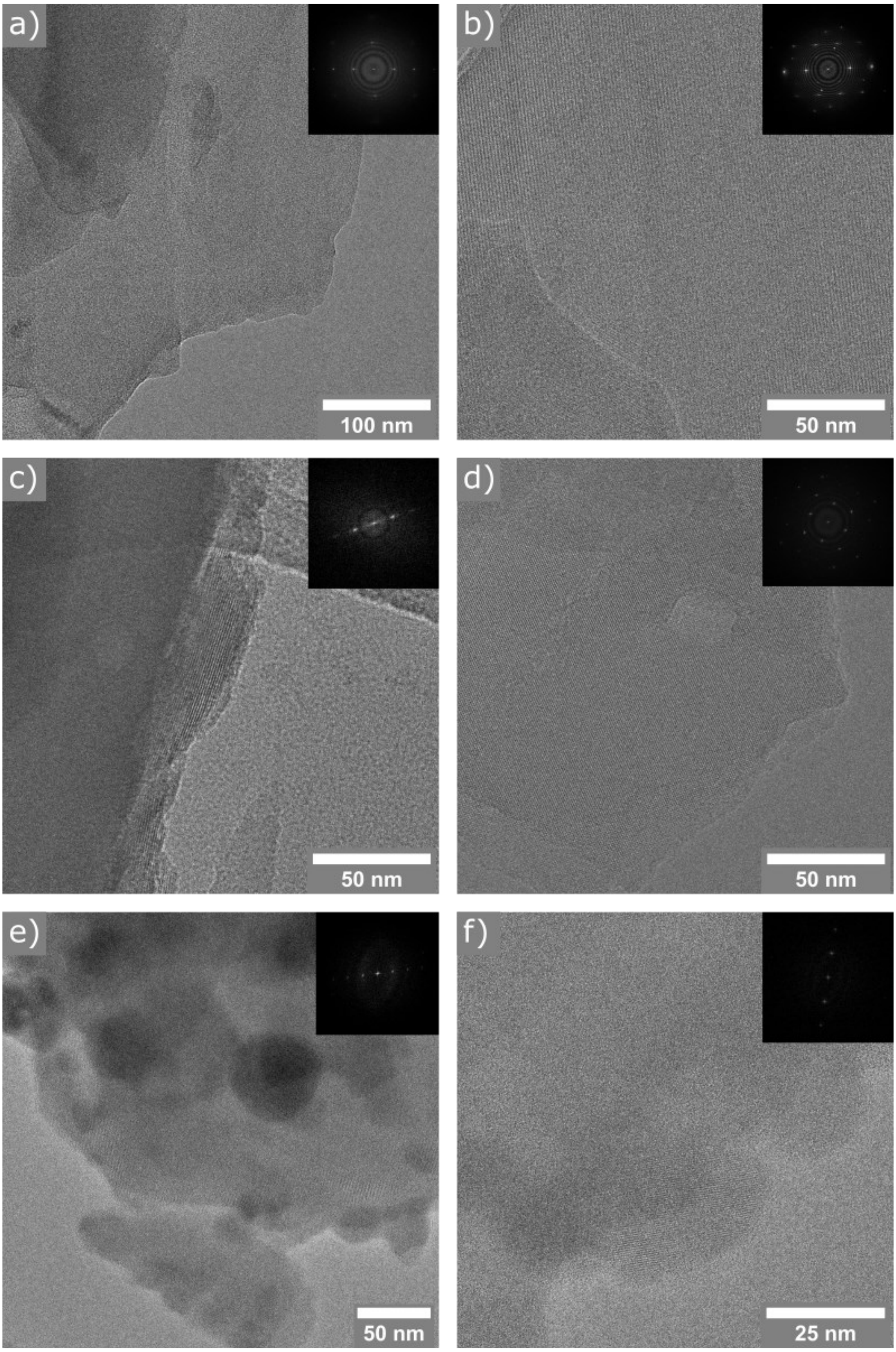

a) 12 M, crystal of a minor IPC-6 phase (d-spacing 10.1 Å); b) 12 M, two overlapping crystals; c) 9 M, lattice fringes corresponding to IPC-2 (d-spacing 11.3 Å); d) 9 M, view along the [101] direction; e) 0.1 ml water, lattice fringes corresponding to IPC-2 (d-spacing 11.2 Å); f) 0.1 ml water, showing a GeO2 nanoparticle impurity.

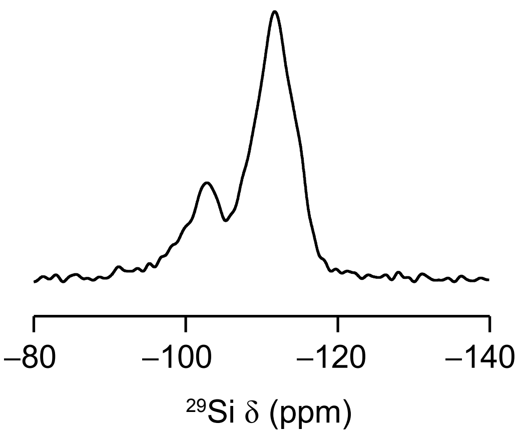

Figure 13: ^{29}Si (9.4 T, 14 kHz) MAS NMR spectrum of calcined UTL, ball milled in 40% $H_2{}^{17}O$ for 30 minutes and dehydrated under vacuum at 120 ℃ overnight.
